# Impact of kidney size on the outcome of diabetic patients receiving hemodialysis

**DOI:** 10.1371/journal.pone.0266231

**Published:** 2022-03-31

**Authors:** Min Wang, Hsin-Chiao Hsu, Mei-Ching Yu, I-Kuan Wang, Chien-Chang Huang, Ming‐Jen Chan, Cheng-Hao Weng, Wen-Hung Huang, Ching-Wei Hsu, Lan-Mei Huang, Frederick W. K. Tam, Tzung-Hai Yen

**Affiliations:** 1 Department of Nephrology, Clinical Poison Center, Kidney Research Center, Center for Tissue Engineering, Chang Gung Memorial Hospital, Linkou, and Chang Gung University, Taoyuan, Taiwan; 2 Division of Pediatric Nephrology, Department of Pediatrics, Chang Gung Memorial Hospital, Linkou, and Chang Gung University, Taoyuan, Taiwan; 3 Department of Nephrology, China Medical University Hospital, Taichung, and China Medical University, Taichung, Taiwan; 4 Department of Immunology and Inflammation, Centre for Inflammatory Disease, Imperial College London, London, United Kingdom; Istituto Di Ricerche Farmacologiche Mario Negri, ITALY

## Abstract

**Introduction:**

Diabetic patients normally have enlarged or normal-sized kidneys throughout their lifetime, but some diabetic uremic patients have small kidneys. It is uncertain if kidney size could have any negative impact on outcome in hemodialysis patients.

**Methods:**

This longitudinal, observational cohort study recruited 301 diabetic hemodialysis patients in 2015, and followed until 2019. Patients were stratified into two subgroups according to their kidney sizes before dialysis, as small (n = 32) or enlarged or normal (n = 269). Baseline demographic, hematological, biochemical, nutritional, inflammatory and dialysis related data were collected for analysis.

**Results:**

Patients with small kidney size were not only older (P<0.001) and had lower body mass index (P = 0.016), but had also higher blood uric acid concentration (P<0.001) compared with patients with enlarged or normal kidney size. All patients received adequate doses of hemodialysis since the Kt/V and urea reduction ratio was 1.7±0.3 and 0.7±0.1, respectively. Patients with small size kidneys received higher erythropoietin dose than patients with enlarged or normal kidney size (P = 0.031). At the end of analysis, 92 (30.6%) patients expired. Kaplan-Meier analysis revealed no survival difference between both groups (P = 0.753). In a multivariate logistic regression model, it was demonstrated that age (P<0.001), dialysis duration (P<0.001), as well as blood albumin (P = 0.012) and low-density lipoprotein (P = 0.009) concentrations were significantly correlated with mortality.

**Conclusions:**

Small kidney size on starting hemodialysis was not related with an augmented risk for death in diabetic patients receiving hemodialysis. Further studies are necessary.

## Introduction

Kidneys are normally enlarged in patients with diabetes mellitus, and both kidneys stay enlarged even in the terminal phase of progressive chronic kidney disease. In a research [1], it was stated that diabetic patients had a 1.723-fold change of having an enlarged kidney. Furthermore, another study [2] confirmed that diabetic patients with large kidneys sufffered progression renal disease deterioration. Additionally, previous study [3] revealed that larger renal length was related to greater odds of cardiovascular complications, and kidney length may serve as an useful biomarker to identify patients at high cardiovascular risk.

Nevertheless, small kidneys could be detected in some diabetic patients with renal insufficiency. It was presented that 81.2% of diabetic patients with renal insufficiency had small kidneys, implicating that these patients suffered ischemic, hypertonic or inflammatory nephropathy accompanying diabetes [4]. Other study [5] suggested that the early renal changes in patients with diabetes mellitus are primarily due to thickening of tubular basement membrane, causing renal hypertrophy. In contrast, many tubulointerstitial insults could produce apoptosis, causing tubular fibrosis and lastly renal atrophy [5]. Moreover, atherosclerosis and associated ischemic insults could reduce kidney perfusion and inducing kidney atrophy [5].

Taiwan is an epidemic area of chronic kidney disease [6]. The motivation for this research was due to an significant, but as yet unsatisfactorily answered question that occurred in numerous diabetic patients receiving hemodialysis at our dialysis unit. The majority of our patients had enlarged or normal kidneys on starting hemodialysis, but certain patients had small kidneys when starting hemodialysis. Hence, this brings up an important question of what the influence of renal size on the mortality outcome of the patients is.

Diabetes accounts for the principal etiology of end-stage kidney disease in most countries, but no effort been made to compare mortality outcome of diabetic hemodialysis patients with and without kidney size, which stimulated our curiosity in the topic. Our previous analysis [7] demonstrated that diabetic patients with small kidney size at the commencement of peritoneal dialysis suffered a greater odds for mortality compared to patients with enlarged or normal renal size. Therefore, we attempted to examine renal size in diabetic patients on starting hemodialysis, and to investigate the correlation of renal size with mortality outcome as well as clinical parameters. Furthermore, these data will be analyzed and interpreted by comparing to previous research on peritoneal dialysis.

## Results

Although the majority of diabetic patients had enlarged or normal kidney size (n = 269, 89.4%) on starting hemodialysis, certain patients suffered from small kidney size (n = 32, 10.6%) on starting hemodialysis (Table 1). The patients aged 58.8 ± 12.1 years, and dialyzed for 4.1 ± 3.3 years. Eight (3.0%) patients with enlarged or normal kidney size received kidney biopsy, and the results showed diabetic nephropathy without any coexisting non-diabetic kidney disease such as glomerulonephritis. None of the patients were on immunosuppressive medications. Hypertension (92.4%) was common in hemodialysis patients. Patients with small kidney size were not only older (65.7 ± 13.7 versus 58.0 ± 11.7 years, P < 0.001), but also had lower body mass index (22.4 ± 2.6 versus 24.0 ± 3.7 kg/m^2^, P = 0.016) than patients with enlarged or normal kidney size. No significant differences noted for other variables.

**Table 1:** Baseline characteristics of diabetic patients receiving hemodialysis stratified by kidney size (n = 301).

Variable	All patients (n = 301)	Patients with enlarged or normal kidney size (n = 269)	Patients with small kidney size (n = 32)	P value
Left kidney, cm	10.2 ± 2.1	10.4 ± 2.0	8.1 ± 1.6	< 0.001***
Right kidney, cm	10.2 ± 1.7	10.5 ± 1.6	8.2 ± 0.6	< 0.001***
Age, year	58.8 ± 12.1	58.0 ± 11.7	65.7 ± 13.7	< 0.001***
Female, n (%)	132 (43.9)	115 (42.8)	17 (53.1)	0.264
Body mass index, kg/m^2^	23.8 ± 3.7	24.0 ± 3.7	22.4 ± 2.6	0.016*
Dialysis duration, year	4.1 ± 3.3	4.1 ± 3.3	4.0 ± 3.4	0.965
Polycystic kidney disease, n (%)	2 (0.7)	2 (0.7)	0 (0)	0.625
Kidney biopsy, n (%)	8 (2.7)	8 (3.0)	0 (0)	0.323
Biopsy-proved diabetic nephropathy, n (%)	8 (2.7)	8 (3.0)	0 (0)	0.323
Biopsy-proved glomerulonephritis, n (%)	0 (0)	0 (0)	0 (0)	1.000
Hypertension, n (%)	278 (92.4)	249 (92.6)	29 (90.6)	0.696
Cardiovascular disease, n (%)	47 (15.6)	40 (14.9)	7 (21.9)	0.302
Duration of diabetes mellitus, year	13.9 ± 7.4	13.9 ± 7.2	13.8 ± 9.2	0.967
Hypoglycemic therapy				0.480
Insulin therapy, n (%)	140 (46.5)	127 (47.2)	13 (40.6)	
Oral hypoglycemic agent, n (%)	161 (53.5)	142 (52.8)	19 (59.4)	
Immunosuppressive medications, n (%)	0 (0)	0 (0)	0 (0)	1.000
Alcohol consumption, n (%)	54 (17.9)	49 (18.2)	5 (15.6)	0.718
Smoking habit, n (%)	77 (25.6)	70 (26.0)	7 (21.9)	0.611
Betel nut chewing, n (%)	26 (8.6)	24 (8.9)	2 (6.3)	0.611

Note

**P < 0.01

***P<0.001.

Table 2 shows that patients with small size kidneys suffered higher blood levels of uric acid (9.2 ± 10.6 versus 6.7 ± 1.5 mg/dL, P < 0.001) than patients with enlarged or normal kidney size. No significant differences found for other variables.

**Table 2:** Laboratory data of diabetic patients receiving hemodialysis stratified by kidney size (n = 301).

Variable	All patients (n = 301)	Patients with enlarged or normal kidney size (n = 269)	Patients with small kidney size (n = 32)	P value
Blood urea nitrogen, mg/dL	67.7 ± 17.7	67.1 ± 17.0	72.5 ± 22.3	0.101
Creatinine, mg/dL	9.4 ± 2.4	9.4 ± 2.4	9.4 ± 2.0	0.999
Uric acid, mg/dL	7.0 ± 3.7	6.7 ± 1.5	9.2 ± 10.6	<0.001***
Sodium, mEq/L	136.6 ± 7.7	136.4 ± 8.1	137.7 ± 2.7	0.373
Potassium, mEq/L	4.8 ± 0.8	4.8 ± 0.8	4.8 ± 0.9	0.797
Chloride, mEq/L	99.3 ± 3.3	99.1 ± 3.3	100.3 ± 2.7	0.057
Calcium, mg/dL	9.3 ± 0.9	9.3 ± 0.9	9.3 ± 0.9	0.870
Phosphate, mg/dL	4.9 ±1.4	4.9 ± 1.4	4.7 ± 1.2	0.410
Bicarbonate, mmol/L	22.4 ± 3.0	22.4 ± 3.1	22.1 ± 2.5	0.682
Fasting glucose, mg/dL	160.3 ± 81.2	162.2 ± 82.7	144.7 ± 66.3	0.249
Glycated hemoglobin, %	7.3 ± 1.9	7.4 ± 1.9	6.9 ± 1.8	0.189
Albumin, g/dL	4.0 ± 0.4	4.0 ± 0.4	4.0 ± 0.3	0.788
Total bilirubin, mg/dL	0.3 ± 0.1	0.3 ± 0.1	0.3 ± 0.1	0.882
Alkaline phosphatase, U/L	78.7 ±30.7	78.2 ± 30.3	82.3 ± 36.1	0.483
Total cholesterol, mg/dL	163.7 ± 36.7	164.8 ± 37.0	154.7 ± 33.7	0.141
High-density lipoprotein, mg/dL	38.8 ± 13.1	39.0 ± 13.2	37.2 ± 12.8	0.465
Low-density lipoprotein, mg/dL	90.8 ± 30.5	91.5 ± 31.0	84.1 ± 25.9	0.194
Triglyceride, mg/dL	177.8 ± 127.3	179.2 ± 125.1	166.0 ± 146.4	0.581
Aspartate aminotransferase, U/L	21.4 ± 14.9	21.1 ± 15.3	23.7 ± 10.8	0.353
Alanine aminotransferase, U/L	16.1 ± 10.1	15.7 ± 9.8	19.3 ± 12.3	0.060
Gamma-glutamyl transferase, U/L	31.7 ± 32.3	31.5 ± 30.6	33.3 ± 44.9	0.755
Iron, ug/dL	64.9 ± 28.9	65.3 ± 28.5	61.7 ± 32.2	0.519
Total iron binding capacity, ug/dL	246.3 ± 45.6	247.3 ± 46.1	238.1 ± 40.7	0.296
Ferritin, ng/mL	428.4 ± 388.2	422.5 ± 390.0	476.9 ± 375.8	0.469
Transferrin saturation, %	26.6 ± 11.5	26.6 ± 11.4	27.1 ± 12.2	0.838
Intact parathyroid hormone, pg/mL	222.9 ± 213.0	221.2 ± 216.2	237.0 ± 186.5	0.693
High sensitivity C-reactive protein, mg/L	8.0 ± 11.9	7.9 ± 11.6	8.9 ± 14.4	0.660
White blood cell count, 10^3^/uL	7.2 ± 3.3	7.3 ± 3.4	6.7 ± 2.3	0.316
Red blood cell count, 10^6^/uL	3.6 ± 0.6	3.6 ± 0.6	3.5 ± 0.4	0.156
Hemoglobin, g/dL	10.4 ±1.5	10.5 ± 1.5	10.3 ± 0.9	0.446
Hematocrit, %	31.9 ± 4.0	32.0 ± 4.1	31.2 ± 2.9	0.293
Mean corpuscular volume, fL	88.6 ± 8.6	88.8 ± 7.3	87.5 ± 16.1	0.427
Platelet count, 10^3^/uL	201.5 ± 64.0	203.8 ± 65.2	182.8±50.1	0.080

Note

*P < 0.05

**P < 0.01.

As shown in Table 3, all patients received adequate doses of hemodialysis since the Kt/V and urea reduction ratio was 1.7 ± 0.3 and 0.7 ± 0.1, respectively. The average residual glomerular filtration rate was 5.0 ± 4.9 mL/min. Patients with small size kidneys received higher erythropoietin dose than patients with enlarged or normal kidney size (20703.7 ± 8193.9 versus 16765.4 ± 8879.5 unit/month, P = 0.031). No significant differences noted for other variables.

**Table 3:** Dialysis related data of diabetic patients receiving hemodialysis stratified by kidney size (n = 301).

Variable	All patients (n = 301)	Patients with enlarged or normal kidney size (n = 269)	Patients with small kidney size (n = 32)	P value
Kt/V	1.7 ± 0.3	1.6 ± 0.3	1.7 ± 0.3	0.441
Urea reduction ratio	0.7 ± 0.1	0.7 ± 0.1	0.8 ± 0.1	0.207
Time-averaged concentration of urea, mg/dL	41.6 ±12.9	41.2 ± 12.7	45.3 ± 14.2	0.085
Normalized protein catabolic rate, g/kg/day	1.1 ± 0.3	1.1 ± 0.3	1.2 ± 0.3	0.068
Erythropoietin, unit/month	17271.7 ± 8874.5	16765.4 ± 8879.5	20703.7 ± 8193.9	0.031*

Note

*P < 0.05

**P < 0.01.

**Fig 1:**
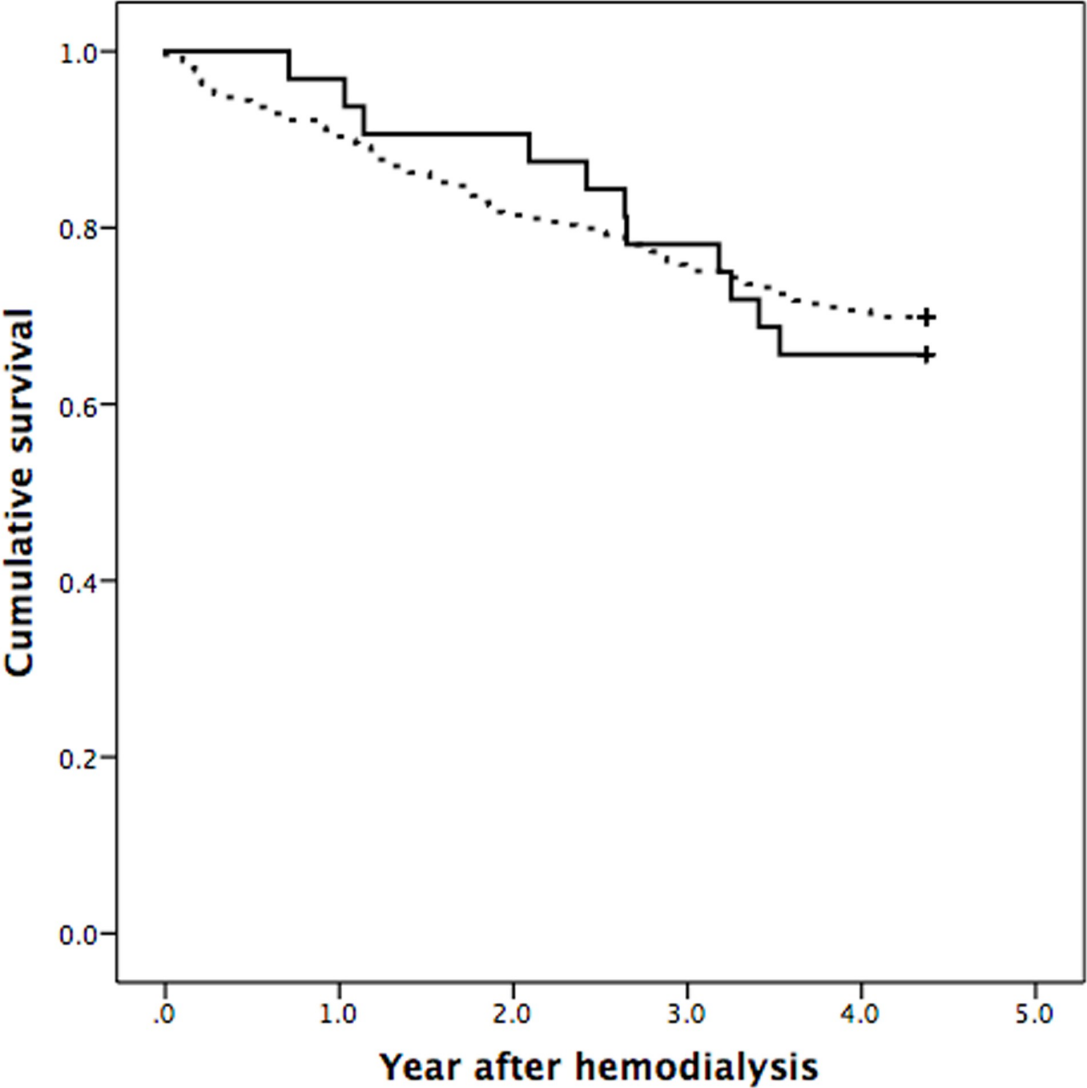
Kaplan-Meier analysis. There was no significant difference in cumulative survival between patients with small kidney size (solid line) and enlarged or normal kidney size (dashed line) (Log-rank test, Chi-Square 0.099, P = 0.753).

A total of 92 of 301 (30.6%) patients died at the end of analysis (Table 4). Cardiovascular disease (17.3%) and infection (13.3) were the main causes of mortality. Kaplan-Meier analysis disclosed no difference in survival between patients with small and enlarged or normal kidney size (Fig 1, Log-rank test, Chi-Square 0.099, P = 0.753).

**Table 4:** Outcomes of diabetic patients receiving hemodialysis stratified by kidney size (n = 301).

Variable	All patients (n = 301)	Patients with enlarged or normal kidney size (n = 269)	Patients with small kidney size (n = 32)	P value
Follow up duration, year	3.6 ± 1.3	3.6 ± 1.4	3.7 ± 1.1	0.696
All-cause mortality, n (%)	92 (30.6)	81 (30.1)	11 (34.4)	0.621
Cardiovascular cause, n (%)	52 (17.3)	46 (17.1)	6 (18.8)	0.817
Infection cause, n (%)	40 (13.3)	35 (13.0)	5 (15.6)	0.687

In a multivariate logistic regression model, it was discovered that age (P < 0.001), dialysis duration (P < 0.001), albumin concentration (P = 0.012) and low-density lipoprotein concentration (P = 0.009) were significantly associated with mortality (Table 5).

**Table 5:** Analysis of mortality using a logistic regression model (n = 301).

Variable	Univariate analysis		Multivariate analysis	
	Odds ratio (95% confidence interval)	P value	Odds ratio (95% confidence interval)	P value
Age (per 1 year increase)	1.054 (1.030–1.078)	< 0.001***	1.072 (1.041–1.104)	< 0.001***
Alanine aminotransferase (per 1 U/L increase)	1.013 (0.986–1.039)	0.353		
Albumin (per 1 g/dL increase)	0.198 (0.092–0.422)	< 0.001***	0.291 (0.111–0.762)	0.012*
Alcohol consumption (yes)	1.178 (0.613–2.262)	0.624		
Alkaline phosphatase (per 1 U/L increase)	0.995 (0.987–1.002)	0.173		
Aspartate aminotransferase (per 1 U/L increase)	1.014(0.985–1.044)	0.360		
Betel nut chewing (yes)	2.591 (0.866–7.752)	0.089		
Bicarbonate (per 1 mmol/L increase)	1.027 (0.947–1.115)	0.519		
Blood urea nitrogen (per 1 mg/dL increase)	1.010 (0.995–1.024)	0.187		
Body Mass Index (per 1 kg/m^2^ increase)	0.942 (0.878–1.010)	0.092		
Calcium (per 1 mg/dL increase)	0.992 (0.747–1.319)	0.957		
Chloride (per 1 mEq/L increase)	0.987 (0.916–1.064)	0.732		
Creatinine (per 1 mg/dL increase)	0.832 (0.745–0.929)	< 0.001***	0.976 (0.832–1.144)	0.765
Dialysis duration (per 1 year increase)	1.121 (1.041–1.206)	0.003**	1.232 (1.124–1.350)	< 0.001***
Duration of onset (per 1 year increase)				
Erythropoietin (per 1 unit/month increase)	1.000 (1.000–1.000)	0.541		
Fasting glucose (per 1 mg/dL increase)	1.002 (0.999–1.005)	0.110		
Female gender (yes)	1.343 (0.820–2.197)	0.241		
Ferritin (per 1 ng/mL increase)	1.000 (1.000–1.001)	0.227		
Gamma-glutamyl transferase (per 1 U/L increase)	1.001 (0.994–1.009)	0.746		
Glycated hemoglobin (per 1% increase)	1.053 (0.923–1.199)	0.441		
Hematocrit (per 1% increase)	1.026 (0.964–1.091)	0.425		
Hemoglobin (per 1g/dL increase)	0.953 (0.805–1.129)	0.579		
High-density lipoprotein (per 1 mg/dL increase)	0.980 (0.962–0.998)	0.029*	0.980(0.958–1.002)	0.072
High sensitivity C-reactive protein (per 1 mg/L increase)	0.972 (0.953–0.992)	0.006**	1.020 (0.997–1.044)	0.090
Hypertension (yes)	1.269 (0.484–3.331)	0.628		
Intact parathyroid hormone (per 1 pg/mL increase)	1.000 (0.999–1.002)	0.605		
Iron (per 1 ug/dL increase)	0.998 (0.990–1.007)	0.721		
Kt/V (per 1 increase)	0.544 (0.249–1.190)	0.127		
Low-density lipoprotein (per 1 mg/dL increase)	1.009 (1.001–1.016)	0.034*	1.012 (1.003–1.021)	0.009**
Mean corpuscular volume (per 1 fL increase)	1.001 (0.973–1.030)	0.963		
Normalized protein catabolic rate (per 1 g/kg/day increase)	0.733 (0.349–1.540)	0.412		
Phosphate (per 1 mg/dL increase)	1.244 (1.032–1.497)	0.022*	1.136 (0.892–1.447)	0.302
Platelet count (per 10^3^/uL increase)	0.998 (0.994–1.002)	0.408		
Potassium (per 1 mEq/L increase)	0.787 (0.571–1.086)	0.145		
Red blood cell count (per 1 10^6^/uL increase)	0.978 (0.634–1.508)	0.918		
Residual glomerular filtration rate (per 1 mL/min increase)	0.981 (0.928–1.037)	0.503		
Small kidney size (yes)	1.216 (0.560–2.638)	0.621		
Smoking habit (yes)	1.045 (0.594–1.838)	0.878		
Sodium (per 1 mEq/L increase)	0.999 (0.967–1.032)	0.948		
Time-averaged concentration of urea (per 1 mg/dL increase)	0.985 (0.966–1.005)	0.132		
Total bilirubin (per 1 mg/dL increase)	2.570 (0.395–16.715)	0.323		
Total cholesterol (per 1 mg/dL increase)	1.006 (1.000–1.013)	0.056		
Total iron binding capacity (per 1 ug/dL increase)	1.003 (0.997–1.009)	0.366		
Transferrin saturation (per 1% increase)	0.994 (0.972–1.017)	0.615		
Triglyceride (per 1 mg/dL increase)	1.001 (0.999–1.003)	0.249		
Urea reduction ratio (per 1 increase)	0.104 (0.004–2.732)	0.175		
Uric acid (per 1 mg/dL increase)	1.129 (0.951–1.341)	0.167		
White blood cell count (per 1 10^3^/uL increase)	1.087 (0.983–1.202)	0.104		

Note

*P < 0.05

**P < 0.01

***P < 0.001.

## Discussion

The analytical results revealed that small kidney size at the starting of hemodialysis was not correlated with augmented mortality in diabetic patients receiving hemodialysis. The research is important because this is the first time kidney size been explored as a potential risk factor for mortality. Previous analysis [7] on diabetic patients found that small kidney size at the starting of peritoneal dialysis is related with increase risk for mortality.

No clear-cut explanations for the absence of a relationship between small kidney size and mortality, but several factors are considered. First is the higher dialysis efficacy and better capacity control of hemodialysis over peritoneal dialysis. Hemodialysis is a much faster and more efficient process than peritoneal dialysis. Therefore, the negative impact of small kidney size (if any) may be compensated by maintenance hemodialysis, but not peritoneal dialysis. Second is the dose of erythropoietin used. Our analysis discovered that patients with small size kidneys received higher erythropoietin dose than patients with enlarged or normal kidney size (P = 0.031). In addition to stimulation of erythropoiesis, erythropoietin also induces multiple pleiotropic effects that are associated with erythropoietin receptor expression in non-erythroid cells [8]. Erythropoietin has an anti-apoptotic activity and exerts a potential neuroprotective, renoprotective and cardioprotective role against ischemia and other kind of damage [9]. Erythropoietin is also involved in angiogenesis, neurogenesis and immune response. It can counteract metabolic changes, vascular and neuronal degeneration, and inflammatory reaction. Nevertheless, it remains uncertain whether the lack of a relationship between small kidney size and mortality could be explained by the higher dose of erythropoietin therapy in this subgroup.

Compared to the previous peritoneal dialysis study [7], the overall mortality rate of diabetic patients was higher in hemodialysis (30.6%) than peritoneal dialysis (16.9%). There was higher overall mortality in the patients on hemodialysis (30.6%) than those on peritoneal dialysis (16.9%), despite the hemodialysis patients are younger (58.8 ± 12.1 years) than peritoneal dialysis patients (69.7 ± 11.6 years). There was no clear explanation. Nevertheless, the mean follow up duration was longer in the hemodialysis (3.6 ± 1.3 year) than peritoneal dialysis (2.4 ± 1.1 year) study. It was shown that the hazards ratio was 1.002 (P = 0.0245) for non-diabetic patients and 1.006 (P = 0.0214) for diabetic patients, suggesting that hemodialysis vintage was a major predictor of mortality in long-term hemodialysis patients, especially diabetic patients [10]. Moreover, the body mass index was lesser in the hemodialysis (23.8 ± 3.7 kg/m^2^) than peritoneal dialysis (25.5 ± 3.7 kg/m^2^) patients. It was unsure if the higher mortality rate could be explained by the lower body mass index in the hemodialysis patients. Previous study from Japanese researcher [11] found that the risk of mortality was increased in dialysis patients with lesser body mass index (< 18.5 kg/m^2^) and diabetes.

Patients with small kidney size were not only older (P < 0.001) and had lesser body mass index (P = 0.016), but had also higher blood uric acid (P < 0.001) than patients with enlarged or normal kidney size. An association of small kidney size with aging is not surprising. With aging, the kidneys undergo physiological changes that are not only the results of normal organ senescence but also of particular diseases (for example atherosclerosis or diabetes) that frequently occur in older people [12]. Macroscopicaally, aging of the kidney is typified by larger medullary volume, smaller cortical volume and cysts [13]. Microscopically, renal aging is typified by a reduced amount of functional glomeruli because of nephrosclerosis (arteriosclerosis, glomerulosclerosis, tubular atrophy and interstitial fibrosis) as well as compensatory hypertrophy of residual nephrons [13]. The association of small kidney size with lower body mass index is also not surprising as kidney size has a direct relationship with body mass index [1,14]. The association of small kidney size with higher blood uric acid level was unclear, and could possibly be explained by poorer uric acid excretion capacity in this subgroup as kidneys eliminate two-thirds of the uric acid load [15]. Nevertheless, hyperuricemia generally develops as a result of the greater production, lesser excretion of uric acid, or a combination of both processes. Therefore, the association needed further exploration.

All the patients received adequate dose of hemodialysis because the Kt/V and urea reduction ratio was 1.7 ± 0.3 and 0.7 ± 0.1, respectively. The KDOQI Clinical Practice Guideline has recommended that a minimal Kt/V of 1.2 was needed to achieve adequate dialysis for patients undergoing thrice weekly hemodialysis [16]. Dialysis Outcomes and Practice Patterns Study reported that a small Kt/V was correlated with greater mortality in hemodialysis patients [17]. National Cooperative Dialysis Study, US Kidney Data System and National Medical Care confirmed that patients treated with greater level of Kt/V or urea reduction ratio enjoyed lower mortality [18–20]. Furthermore, Owen et al [21] reported a negative relationship of higher dialysis does and lower mortality in different race-sex subgroups. The negative correlation was less steep in Kt/V of greater than 0.9 [22]. According to Hemodiaysis study (HEMO study), female hemodialysis patients were randomly assigned to higher (Kt/V 1.7 ± 0.1) and standard (Kt/V 1.3 ± 0.1) subgroup, and the former had longer survival (hazard ratio = 0.81, P = 0.02) in subgroup analysis [23,24]. Kimata et al [17] analyzed the relation between all-cause mortality and patient-level Kt/V in their total sample and gender. Hazard ratio which compared Kt/V ≥ 1.6 to Kt/V < 1.2 showed 0.36 for women and 0.68 for men. In Japan, large part of male hemodialysis patients with low Kt/V had 26% greater mortality than those with Kt/V ≥ 1.2.

The patients aged 58.8 ± 12.1 years. The age of starting hemodialysis in this study was approximate to the Japanese study (60.5 ± 10.2 years) as reported by Tomoaki Morioka et al [25], but younger than Israel study (67.6  ±  9.4 years) as reported by Noa Tsur et al [26]. The age of starting hemodialysis of one study in Korea was 58.1 ± 14.0 (56.4% diabetes), while the age of starting hemodialysis in another study from United States was 64.7 ± 14.5 (46.7% diabetic) [27,28]. In general, the age of starting hemodialysis of this study was not much different from other countries.

A total of 92 of 301 (30.6%) patients died at the end of analysis (Table 4). The 1-, 2-, 3-, and 4-year mortality rates of 32 patients with small kidney size were 3.1%, 9.4%, 21.9% and 34.4%, respectively (Fig 1). The 1-, 2-, 3-, and 4-year mortality rates of 269 patients with enlarged or normal kidney size was 9.7%, 18.6%, 24.9% and 30.1%, respectively (Fig 1). The mortality rates of this study were lower than United States Kidney Data System report, for which the adjusted 3-year and 5-year mortality rate was 43% and 58%, respectively [29]. Furthermore, the UK Renal Registry 20th Annual Report showed that the 1-year mortality for diabetic dialysis patients aged below and above 65 years was 15.4% and 23.1%, respectively [30]. Data from Korean Society of Nephrology presented a 1-year and 3-year mortality rate of 7.6% and 28.4% in diabetic hemodialysis patients [31]. Moreover, the 2013 statistics report of The Japanese Society for Dialysis Therapy revealed 12.4% and 40.2% for 1-year and 5-year mortality rate [32]. Based on the annual report in Singapore, the 1-year and 5-year mortality was 11% and 46.7%, respectively for diabetic hemodialysis patients [33]. Therefore, the mortality data of this study were comparable to other countries.

Compared to the previous peritoneal dialysis study [7], the causes of mortality were dissimilar among patients receiving hemodialysis and peritoneal dialysis. In hemodialysis patients, the causes of mortality were cardiovascular (17.3%), followed by infection (13.3%). In the peritoneal dialysis patients, the causes of mortality were infection (14.5%), followed by cardiovascular (2.4%). The data was unsurprising as patients with peritoneal dialysis were prone to infection [34]. Our past analysis [35] also indicated that bacteremia rate was 7.63 per 100 patient-years in hemodialysis compared with 3.56 per 100 patient-years in peritoneal dialysis. The augmented risks of *cardiovascular mortality in hemodialysis patients* can be accounted for by the high prevalence of traditional and nontraditional factors for cardiovascular risk [36]. Apart from traditional risk factors, patients undergoing hemodialysis were subjected to many non-traditional risk factors for instance anemia, disorders of bone and mineral metabolism, inflammation, etc.

In a multivariate logistic regression model, it was confirmed that age (P < 0.001), dialysis duration (P < 0.001), and albumin (P = 0.012) and low-density lipoprotein (P = 0.009) were correlated with greater risk of death. Many investigators have proved a positive relationship of older age and mortality risk. One study reported that age was a significant predictor for mortality after analyzing 556 Japanese patients receiving less than 10 years of hemodialysis [37]. Another study in United States indicated that older age was one of five independent predictors of mortality, with a hazard ratio of 1.36 in 10-year interval [38]. A meta-analysis study [39] also demonstrated that longer hemodialysis duration increased the risk of cardiac death. Serum albumin level has been recognized as a significant predictor of death in hemodialysis population. For example, serum albumin was confirmed to possess predictive value of mortality risk for hemodialysis patients according to one study from United States [40]. The stronger association of lower albumin levels with higher risk of death was observed not only in the Dialysis Outcomes and Practice Patterns Study (DOPPS) involving 7 countries [41], but also in another DOPPS of 40,950 hemodialysis patients across 12 countries [42]. Our previous analysis [43] also noted that normalized protein catabolic rate ≧1.2 g/kg/day with albumin < 4 g/dL and normalized protein catabolic rate < 1.2 g/kg/day with albumin < 4 g/dL were significant predictors for mortality in patients receiving maintenance hemodialysis. Finally, the association between low-density lipoprotein and mortality is not surprising since dyslipidemia is a firmly established traditional risk factor for cardiovascular events in the dialysis patients [44].

This research is restricted by low sample size and short duration of follow-up. Moreover, this research is restricted by absence of protocol renal biopsies in the diabetic patients. The risk /benefit ratio of renal biopsies in diabetic patients, particularly in the patients with small kidney size has been an important factor. Only eight (3.0%) patients with enlarged or normal kidney size underwent renal biopsy, but none of the patients with small kidney size received biopsy (Table 1). The diabetic patient who became end-stage kidney disease may combine with several etiologies or even other type of glomerulonephritis, which while no kidney biopsy proved, we could just call the patient also with diabetes mellitus. While the patient became end-stage kidney disease without enlargement kidney size, which cannot exclude just because combine with other comorbidity. Further research is needed.

## Conclusions

Small kidney size on starting hemodialysis was not related with an augmented risk for death in diabetic patients receiving hemodialysis. Instead, age, hemodialysis duration, albumin and low-density lipoprotein levels were the significant predictor of mortality in the hemodialysis patients.

## Materials and methods

### Ethical statement

The study design was performed according to the criteria set by the declaration of Helsinki and was approved by the Medical Ethics Committee of Chang Gung Memorial Hospital, Linkou, Taiwan. Because this study was a retrospective review of existing data, Institutional Review Board approval was obtained, but without specific informed consent from the patients. The Institutional Review Board of Chang Gung Memorial Hospital specifically waived the need for consent. The institutional review board numbers was 202000663B0.

### Patients

**Fig 2:**
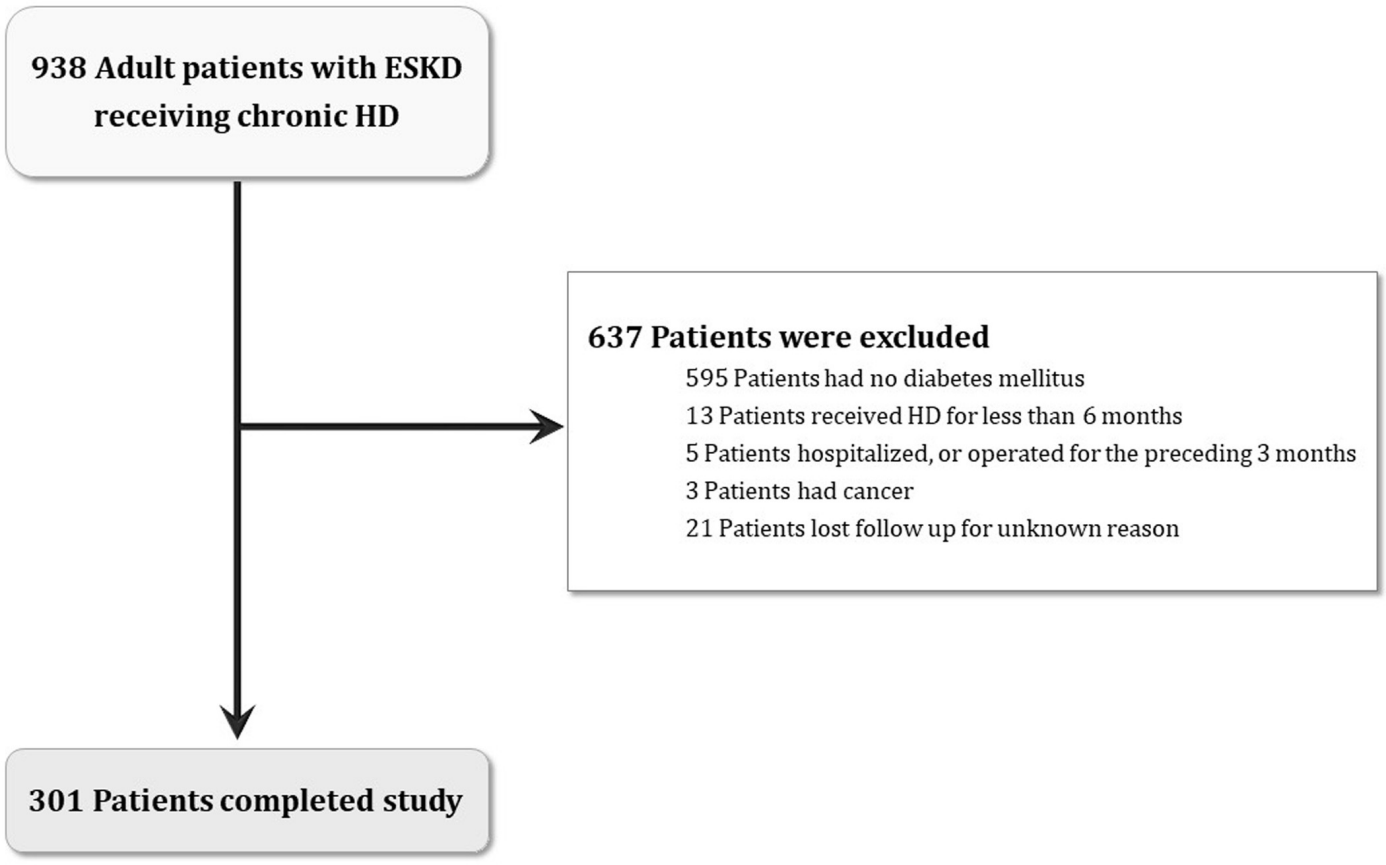
Flow chart. Diagram shows the enrolment and status of patients. ESKD end-stage kidney disease, HD hemodialysis.

This longitudinal, observational cohort study enrolled 301 diabetes patients undergoing chronic hemodialysis at Chang Gung Memorial Hospital (Fig 2). Oral hypoglycemic agent or insulin therapy was used to control blood sugar, and their blood glucose level as well as glycated hemoglobin level were regularly monitored. Patients’ demographics such as age, sex, body mass index, hypertension, dialysis duration, etc were recorded. Smoking habit, alcohol consumption and betel nut chewing habit were also traced. Patients with hypertension took antihypertensive medications regularly. Laboratory and dialysis-related data were recorded, and mortality data were collected for analysis.

### Patient group

These 301 diabetic patients were categorized into two subgroups according to their renal size at the starting of hemodialysis, as enlarged or normal (n = 269) or small (n = 32) kidney size. All patients received ultrasonographic examination, and small kidney was defined as a renal length of below 9.0 cm [4].

### Inclusion and exclusion criteria

All adult diabetic patients undergoing chronic hemodialysis at Chang Gung Memorial Hospital in 2015 were recruited into this research. Patients without diabetes mellitus, patients younger than 18 years, patients received less than 6 months of chronic hemodialysis, patients received hospitalization or operation within 3 months prior to this study as well as patients with malignancy were rejected from research involvement.

### Hemodialysis prescription

All patients were on maintenance hemodialysis prescription, each hemodialysis treatment lasted four hours and was performed thrice a week. Hemodialysis was performed with single-use hollow-fibre artificial kidneys fitted with modified cellulose-based, polyamide or polysulfone membranes. The dialysate was a ionic composition with bicarbonate-based buffer, and a reverse osmosis filter system had been applied for water treatment.

### Laboratory

The data was the last laboratory findings before these patient being began on chronic hemodialysis program. Blood concentrations of albumin, urea nitrogen, creatinine and transferrin saturation as well as normalized protein catabolic rate were determined and used as nutritional markers. High sensitivity C reactive protein was measured and used as an inflammatory marker. Daugirdas formula was used to calculate the Kt/V. Normalized protein catabolic rate was calculated with validated equations, and normalized to patients’ body weight. Blood concentrations of calcium, phosphate and as well as parathyroid hormone were examined. Other laboratory analysis, including complete blood cell and biochemistry were measured by automated and standardized methods.

### Statistics

Continuous parameters were reported as means ± standard deviation, whereas categorical parameters as numbers and percentages in parenthesis. Student’s t test was applied to examine the quantitative parameters, and Chi-square or Fisher’s exact test, for categorical parameters. Univariate logistic regression analysis was perrformed to analyze the predictors for mortality, and multivariate logistic regression analysis, to spot significant related factors. A P value of lower than 0.05 is statistically significant. Data were analyzed with IBM SPSS Statistics Version 20.0.
